# Pilot phenotype and natural history study of hereditary neuropathies caused by mutations in the *HSPB1* gene

**DOI:** 10.1016/j.nmd.2016.10.001

**Published:** 2017-01

**Authors:** Alexander M. Rossor, Jasper M. Morrow, James M. Polke, Sinead M. Murphy, Henry Houlden, INC-RDCRC, Matilde Laura, Hadi Manji, Julian Blake, Mary M. Reilly

**Affiliations:** a MRC Centre for Neuromuscular Diseases, National Hospital for Neurology and Neurosurgery, UCL Institute of Neurology, Queen Square, London, WC1N 3BG, UK; b Department of Neurogenetics, The National Hospital for Neurology and Neurosurgery, UCL Institute of Neurology, London, UK; c Department of Neurology, Adelaide & Meath Hospitals Incorporating the National Children's Hospital, Tallaght, Dublin, Ireland; d Academic Unit of Neurology, Trinity College Dublin, Ireland; e Department of Clinical Neurophysiology, Norfolk and Norwich University Hospital, UK

**Keywords:** Distal hereditary motor neuropathy, Charcot–Marie–Tooth disease, HSPB1, Neuromuscular disease, Peripheral neuropathy

## Abstract

Mutations in *HSPB1* are one of the commonest causes of distal Hereditary Motor Neuropathy (dHMN). Transgenic mouse models of the disease have identified HDAC6 inhibitors as promising treatments for the condition paving the way for human trials. A detailed phenotype and natural history study of *HSPB1* neuropathy is therefore required in order to inform the duration and outcome measures of any future trials. Clinical and neurophysiological data and lower limb muscle MRI were collected both prospectively and retrospectively from patients with mutations in *HSPB1*. The natural history was assessed by recording the weighted Charcot–Marie–Tooth Examination Score (CMTES) at annual intervals in a subset of patients. 20 patients from 14 families were recruited into the study. The average age of onset was in the 4th decade. Patients presented with a length dependent neuropathy but with early ankle plantar flexion weakness. Neurophysiology confirmed a motor neuropathy but also showed sensory nerve involvement in most patients. Cross sectional muscle MRI revealed soleus and medial gastrocnemius fat infiltration as an early signature of mutant *HSPB1* disease. In this study neither semi quantitative muscle MRI, the CMTES nor neurophysiology were able to detect disease progression in HSPB1 neuropathy over 1 or 2 years. Further studies are therefore required to identify a suitable biomarker before clinical trials in HSPB1 neuropathy can be undertaken.

## Highlights

• Mutations in *HSPB1* result in a motor predominant neuropathy.
• The mean age of disease onset was in the 4th decade.
• HSPB1 neuropathy is characterised by early plantar flexion weakness.
• Muscle MRI demonstrates selective denervation of gastrocnemius and soleus.

## 1 Introduction

Charcot–Marie–Tooth disease (CMT) is the most common genetic neuromuscular disease with a population prevalence of 1 in 2500. The distal hereditary motor (dHMN) and hereditary sensory neuropathies refer to forms of CMT in which the disease burden falls on either motor or sensory nerves respectively [1]. CMT and related disorders are a common and genetically heterogenous group of diseases for which more than 80 causative genes have now been described [2]. Mutations in the small heat shock protein, *HSPB1*, although very rare, are the commonest cause of dHMN and have also been reported to cause CMT2 [3], [4]. In this study we aimed to characterise the phenotype and natural history of a large, single centre cohort of patients with mutations in *HSPB1*.

*HSPB1* is a member of the family of small heat shock proteins. Heat shock proteins (HSPs) are molecular chaperones that are classified according to their molecular weight. HSPs were originally identified as proteins that were induced following heat shock and prevented or reversed the misfolding of cellular proteins [5]. Why mutations in such a ubiquitously expressed protein should result in an isolated neuropathy is not clear.

Mutations in *HSPB1* were first identified as a cause of autosomal dominant dHMN and CMT2 in 2004 [6] following which mutations have been described spanning all regions of the protein [7]. Four mutant *HSPB1* transgenic mouse models of dHMN have now been developed [8], [9], [10] and in 2011, d'Ydewalle et al. observed that treatment with a selective HDAC6 inhibitor successfully reversed the clinical phenotype of both S135F and P182L transgenic mice [8]. Further studies involving the use of HDAC 6 inhibitors in other models of inherited and chemotherapy induced neuropathy have revealed promising pilot results [11], [12] paving the way for future clinical trials in patients. Trials of novel therapies in rare diseases, however, require data on the detailed phenotype and natural history of the disease with which to inform appropriate trial design. In this paper we summarise the clinical, neurophysiological and radiological phenotype of a large, single centre cohort of 20 patients from 14 families with mutations in *HSPB1* followed up over a range of 1–10 years by the same investigator (MMR).

## 2 Methods

Patients were recruited from the inherited neuropathy clinic at the National Hospital for Neurology and Neurosurgery, London. This study was approved by The National Hospital for Neurology and Neurosurgery (NHNN) Research Ethics Committee/Central London REC 3 09/H0716/61.

The *HSPB1* mutations were identified by either Sanger sequencing, whole exome sequencing (WES) or the use of CMT2 disease specific next generation sequencing panels.

### 2.1 Clinical assessment

Neurological history, examination, and nerve conduction study were performed in all patients. In a subset of patients (patients 1 (ii), 8, 11, 12, 13 (i), 13 (ii), 13 (iv), 14 (ii)), the Rasch modified CMT examination score (hereto referred to as the weighted CMTES) was measured prospectively [13]. As nerve conduction studies were not always performed at the same time as the clinical examination, only the weighted CMTES rather than the Rasch modified CMTNSv2 was calculated. Patients were evaluated annually when possible.

### 2.2 Lower limb muscle MRI

Six out of 20 patients were scanned at 3 Tesla (Siemens TIM Trio, Erlangen, Germany) in a supine position with surface array coils to receive the signal from the thighs and calves of both limbs. Patients were scanned with a clinical imaging protocol comprising T1 weighted axial imaging and axial STIR imaging as previously described [14]. Muscle MRI scans were assessed for normal and abnormal muscle bulk and for normal and abnormal signal intensity within the different muscle groups. All muscle MRI scans were assessed by an independent observer (JM) and scored according to the 2002 Mercuri classification [15]; a six-point semi-quantitative scale with 0 = normal muscle, 4 = muscle completely replaced by fat. The following muscles were scored bilaterally: rectus femoris, vastus intermedius, vastus lateralis, vastus medialis, semimembranosus, semitendinosus, biceps femoris, adductor magnus, gracilis and sartorius in the thigh; tibialis anterior, peroneus longus, medial gastrocnemius, lateral gastrocnemius, soleus and tibialis posterior in the calf. The mean Mercuri scores for the calf and thigh were also calculated as an overall measure of disease severity on MRI. Serial MRI scans were obtained for two patients. JM assessed these blinded to the chronological order of these two sets of MRI scans.

### 2.3 Statistical analysis

All statistical analyses were performed using Microsoft Excel (paired t-test) and SPSS version 14.0 (Spearman's rank coefficient and Chi squared analysis).

## 3 Results

### 3.1 Mutation analysis

Sanger sequencing of *HSPB1* identified two previously unreported mutations, S135Y and P182A. The S135Y mutation was identified in a sporadic Somalian patient and is likely to be pathogenic as the S135F mutation (i.e. substitution of the same amino acid) is the commonest published pathogenic mutation in HSPB1 [6], [7]. DNA was not available from the patient's siblings or parents.

The P182A mutation is likely to be pathogenic as it was found to segregate with the disease in all six family members for whom DNA was available (five affected and one unaffected). In addition, two different missense mutations at the same amino acid (182) have previously been reported to cause dHMN [6], [16].

The P182A mutation in family 14 was initially missed by Sanger sequencing of the *HSPB1* gene in two affected family members and subsequently identified using whole exome sequencing. The reason for this false negative result was identified as being due to a 4-bp insertion in intron 2 on the same allele as the P182A mutation (*HSPB1* comprises 3 exons with the P182A mutation residing in exon 3). The 4-bp insertion (GGTG) occurs within a G/C rich region, 3xGGTG repeat sequence, and is present on dbSNP (rs30617181). The additional GGTG repeat prevented this allele from being amplified in the original PCR causing the sequencing to appear normal. Use of a proof reading polymerase confirmed the P182A mutation identified using WES as well as insertion of the GGTG intronic sequence.

### 3.2 Clinical presentation

**Fig. 1:**
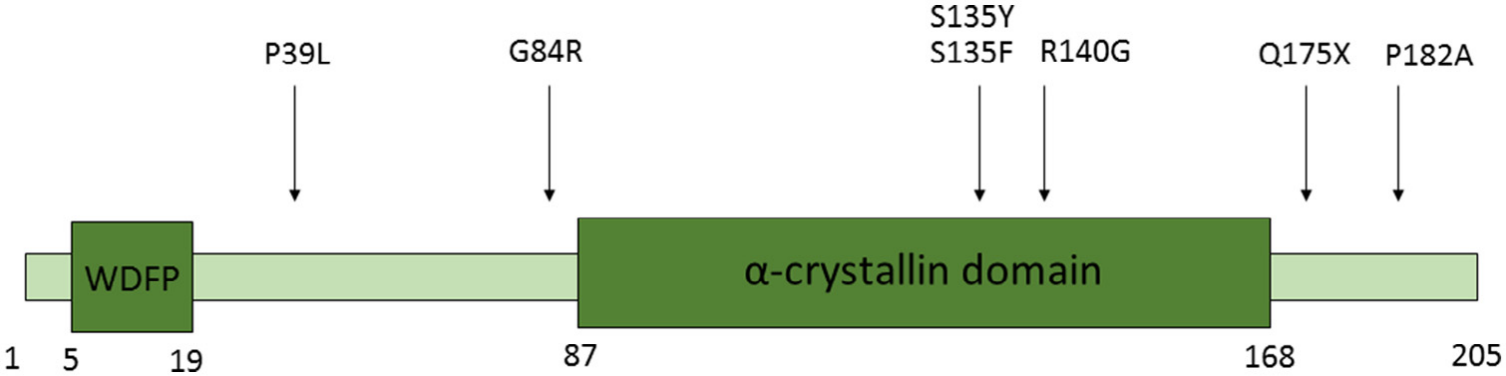
A schematic diagram of the HSPB1 protein indicating the mutations identified in this study relative to the hydrophobic N terminal WDFP and the conserved alpha-crystallin domains.

The average age of onset of the disease was in the 4th decade although this ranged from the second to the 6th decade (see Table 1.) There was no clear genotype–phenotype correlation; the age of onset was in the second decade for families with both the S135Y and P182A mutations i.e. both within and outside of the alpha crystallin domain (See Fig. 1). Within members of the same family the age of onset was often similar.

Prominent ankle plantar flexion weakness was a common clinical feature unlike most other forms of CMT (see Table 2). Nevertheless, whilst in 8 out of 19 patients, ankle plantar flexion was as-weak as ankle dorsiflexion, in 10 patients, ankle dorsiflexion was weaker than ankle plantar flexion and in only one patient was ankle plantarflexion weaker than dorsiflexion.

The pattern of lower limb weakness was symmetrical in the majority (18/20) of patients. Proximal lower limb weakness was present in 8 out of 20 patients although the neuropathy followed a length dependent pattern in all patients. Reflexes were usually preserved, or even brisk with the exception of the ankle jerks which were absent. Plantar responses were either mute or flexor.

No patients reported delayed walking (>15 months). Five of out 20 patients had collapsed foot arches and no patient had pes cavus. Scoliosis was observed in one patient but this was not severe enough to warrant surgical treatment. No patient needed corrective foot surgery. In five patients in whom information was available the mean interval between diagnosis and the use of ankle foot orthoses was 15 years. All patients remained ambulant. One patient required the use of a walking stick 24 years after the onset of his symptoms and uses a wheelchair intermittently.

### 3.3 Neurophysiology

Neurophysiological data were collected both retrospectively and prospectively from study participants. Nerve conduction studies and EMG were performed in all 20 patients included in this study. Follow up studies were performed in 11 patients over a range of 1–10 years. Electromyography demonstrated length dependent changes of chronic denervation defined by large polyphasic motor units of increased duration and a reduced interference pattern in all patients in the study. Spontaneous activity in the form of positive sharp waves and frequent fibrillation potentials was present in six out of 20 patients.

There was no significant change in any of the parameters over an average 1 year period (Median CMAP = −0.12 mV; Ulnar CMAP = −0.238 mV; Peroneal CMAP = +0.092 mV; Radial SAP = +1.36 µV; Median SAP = −0.90 µV). The largest change was of the sural SNAP which on average deteriorated by −1.8 µV per year. Sensory involvement defined as a reduction in the lower limb sensory nerve action potentials was present in most patients (15/20) and did not appear to show any correlation with the genotype. Even within members of the same family, the degree of sensory involvement was variable (see supplementary Table S1).

### 3.4 Muscle MRI

**Fig. 2:**
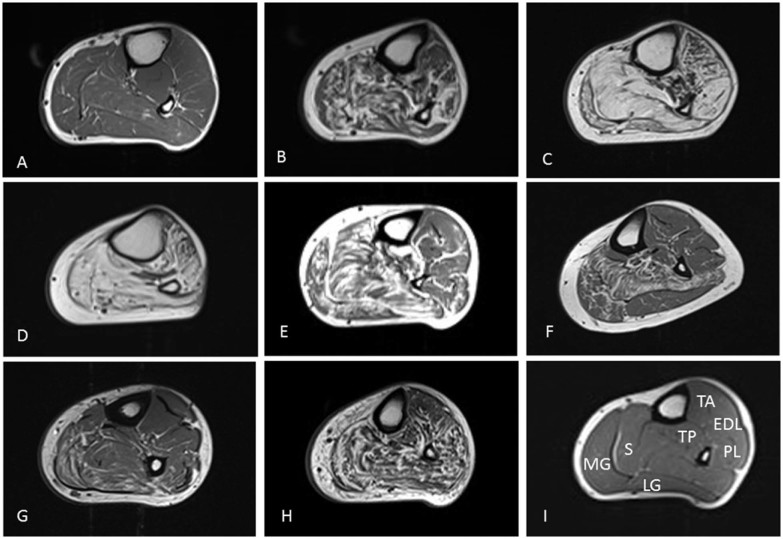
T1 weighted axial MRI images of the left lower limb muscles at mid-calf level in patients with mutations in *HSPB1*. Denervated muscle shows fat infiltration (white). A = patient 2 (Mercuri score = 6), B = patient 7 (41), C = patient 8 (48), D = patient 11 (46), E = patient 12 (34), F = patient 13 (i) (22), G = patient 13 (ii) (20), H = patient 13 (iv) (41), I = Healthy volunteer. Abbreviations: MG = Medial Head of Gastrocnemius, LG = Lateral Head of Gastrocnemius, S = Soleus, TP = Tibialis Posterior, TA = Tibialis Anterior, EDL = Extensor Digitorum Longus, PL = Peroneus Longus.

**Fig. 3:**
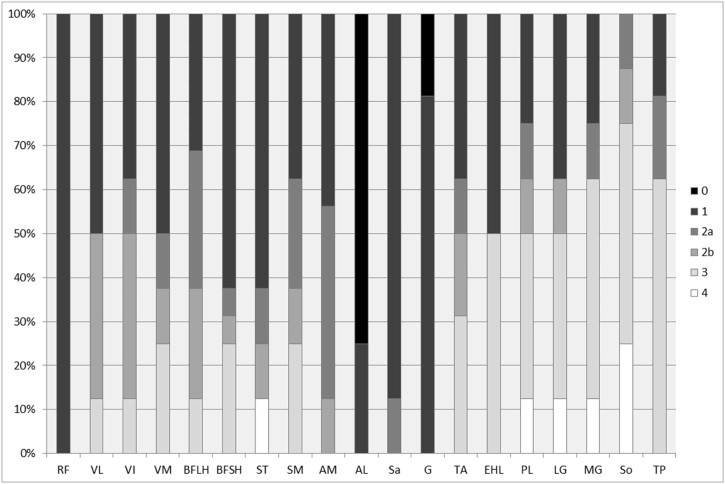
Frequency of Mercuri grades in thigh and calf muscles of HSPB1 patients at baseline (n = 8). The y axis represents the percentage of patients with a particular Mercuri score for each muscle. At the level of the thigh there is sparing of Rectus Femoris and Adductor Longus. At the level of the calf the soleus, medial and lateral gastrocnemius and peroneus longus muscles are more severely affected. RF = Rectus Femoris, VL = Vastus Lateralis, VI = Vastus Intermedius, VM = Vastus Medialis, BFLH = Biceps Femoris Long Head, BFSH = Biceps Femoris Short Head, ST = Semitendinosus, SM = Semimembranosus, AL = Adductor Longus, AM = Adductor Magnus, Sa = Sartorius, G = Gracilis, MG = Medial Head of Gastrocnemius, LG = Lateral Head of Gastrocnemius, So = Soleus, TP = Tibialis Posterior, TA = Tibialis Anterior, EHL = Extensor Hallucis Longus, PL = Peroneus Longus.

Fat infiltration is a key feature of chronically denervated muscle. This can be visualised using muscle MRI in which fat has high signal intensity relative to healthy muscle on T1 weighted sequences. To date, there have been two published reports of muscle MRI in families with mutations in *HSPB1* [17], [18]. Both reports describe fatty infiltration of the medial gastrocnemius and soleus muscles. In this study, muscle MRI was performed in eight individuals from six families with mutations in *HSPB1*. A wide spectrum of fatty infiltration was seen at the calf level, from minimal fatty streaking (Fig. 2A) to near total fatty replacement of all lower leg muscles (Fig. 2D). In those with intermediate levels of involvement, medial gastrocnemius and soleus appeared more affected than the anterior and lateral compartments (Fig. 2E, F and Fig. 3). Significant correlation was seen between ankle dorsiflexion strength and Mercuri grade (rho = −0.9, p < 0.0001), and ankle plantarflexion strength and Mercuri grade (rho = −0.8, p < 0.05). There was no correlation between the degree of fatty infiltration as determined by the Mercuri scores of the calf and thigh and the overall severity of the neuropathy as determined by the CMTES (Calf, rho = 0.7, p = 0.1, n = 6/thigh, rho = 0.6, p = 0.2, n = 6). In addition, there was no correlation between the degree of fatty infiltration of the calf and thigh and the age of examination of the patient (Calf, rho = 0.29, p = 0.76, n = 8/thigh, rho = 0.30, p = 0.76, n = 8).

### 3.5 Prospective natural history data

Ten patients with mutations in *HSPB1* were entered into a natural history study. Eight patients underwent follow up at one year, seven patients follow up at two years and four patients, follow up at three years. There was no significant difference in the weighted CMTES at one (p = 0.7), two (p = 0.64) and three years (p = 0.2, paired t-test, see Table 3). It was not possible to calculate the weighted CMTES retrospectively as old clinical examinations did not include quantitative vibration testing. There was no correlation between the age at time of examination and either the CMTES (rho = 0.64, p = 0.91, n = 6) or weighted CMTES (rho = 0.64, p = 0.91, n = 6).

**Fig. 4:**
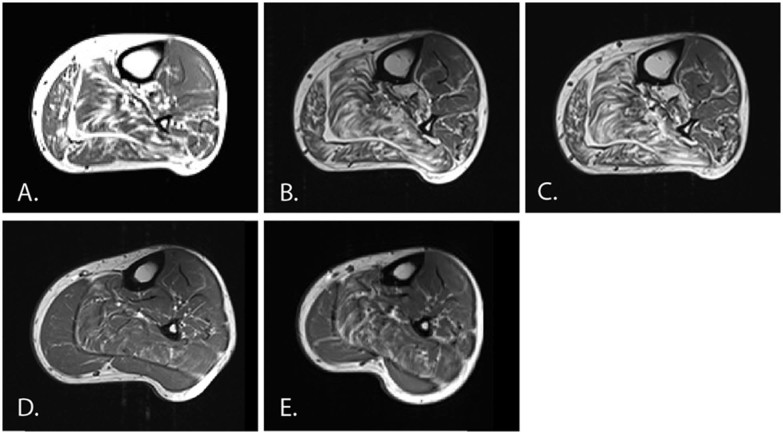
T1 weighted axial MRI images of the lower limbs at baseline, one and two years. A–C are from patient 12 and D–E are from patient 13 (ii). The CMTES and mean calf/thigh Mercuri scores for each image (in parenthesis) are as follows; A, Year 0 (3, 3/1.2); B, Year 1 (7, 3/1); C, Year 2 (3, 3/1); D, Year 0 (0, 0.4/0); E, Year 1 (0, 0.5/0).

To determine whether semi quantitative assessment of calf muscle MRI was sensitive enough to detect disease progression over 1–2 years, muscle MRI was performed at yearly intervals in two patients with dHMN, one with a R140G mutation and the other with a Q175X mutation. Direct visual inspection of the serial MRI scans did not reveal a difference over a 1 or 2 year period. Semi-quantitative assessment also revealed no change over 1 or 2 years (see Fig. 4).

## 4 Discussion

In this study we have described the clinical phenotype of the largest single centre cohort of patients with mutations in *HSPB1* and reported prospective clinical data for a subset of patients followed up over four years. This study includes patients with mutations in *HSPB1* spanning all regions of the HSPB1 protein. Unlike the most common form of CMT, CMT1A, patients with mutations in *HSPB1* had a later age of onset (average 40). Another common feature in our cohort was early plantar as opposed to ankle dorsiflexion weakness which often resulted in symptoms of poor balance. The reason for the early involvement of the soleus and gastrocnemius muscles is unclear but may give important clues to the pathogenesis of the disease.

The study includes detailed prospective and retrospective neurophysiological data for all 20 patients included in the study and in whom a large number underwent repeated investigation over a 10 year follow up period. In the majority of cases the neurophysiology was performed by the same physician (JB). In 6 out of 10 individuals with a recordable sural SAP and in whom there was a follow up nerve conduction study, there was a decline in the sural SAP. In 6 out of 10 individuals with only baseline neurophysiology, the sural SAPs were reduced. This implies that sensory nerves appear to be affected to some degree in most patients with mutations in *HSPB1*.

Although this is the largest natural history study to date, the number of patients followed up was small and as a result no firm conclusions can be reached as to the efficacy of the CMTES, muscle MRI and neurophysiology for detecting change in a larger group of patients.

Qualitative muscle MRI of the lower limbs was performed in eight patients with mutations spanning the entire length of *HSPB1*. The MRI findings correlated with the clinical phenotype showing early signs of denervation in the gastrocnemius and soleus muscles. Follow-up lower limb MRI was obtained in only two patients and was unable to detect change over a 1 or 2 year period using the semi-quantitative Mercuri score. This is perhaps unsurprising as the Mercuri score was intended for pattern description as opposed to longitudinal analysis. It was noted that significant fat infiltration may be seen in muscles with normal bedside strength assessment (e.g. Fig. 2F and G; subjects with normal ankle strength) suggesting MRI may be more sensitive to early disease than clinical assessment.

The development of transgenic mouse models of mutant *HSPB1* neuropathy and the encouraging preclinical studies of HDAC6 inhibitors in *HSPB1* and *GARS* neuropathy have highlighted the need for robust natural history with which to guide future trial design. Unfortunately, our study has failed to show that semi quantitative muscle MRI, the CMTES or neurophysiology are able to detect disease progression in HSPB1 neuropathy. Further studies are therefore required to identify a suitable biomarker before clinical trials in HSPB1 neuropathy are undertaken. HSPB1 related neuropathies, however, are rare and any future prospective trial will require patients to be recruited from multiple centres in order for statistically significant changes in clinical and imaging outcome measures to be identified.

## Figures and Tables

**Table 1:** A summary of the patients included in the study, their mutation, age of onset and country of origin.

Family	No. affected	Inheritance	Mean age at onset/year	Origin	Mutation
1	2	AD	40	England	Pro39Leu
2	1	Sporadic	30	England	Gly84Arg
3	2	AD	55	England	Gly84Arg
4	1	AR	40	Pakistan	Leu99Met
5	1	Sporadic	20	Somalia	Ser135Tyr
6	4	AD	20	Poland	Ser135Phe
7	6	AD	15	Canada	Ser135Phe
8	2	AD	30	Gujurat	Arg140Gly
9	1	AD	40	Gujurat	Arg140Gly
10	2	AD	30	Gujurat	Arg140Gly
11	2	AD	44	Gujurat	Arg140Gly
12	1	AD	40	India	Arg140Gly
13	5	AD	30	England	Gln175X
14	11	AD	13	Ireland	Pro182Ala

**Table 2:** A summary of the baseline clinical characteristics of patients with mutations in HSPB1.

Patient	Age at examination	ADF	APF	FDIO	APB	Proximal UL/LL weakness
1 (i)	79	2/4-	4-/4	4/4	4/4	N/N
1 (ii)	69	0/0	1/0	3/3-	4/4	N/Y
2	46	5/5	5/5	5/5	5/5	N/N
3	68	2/2	3/3	5/5	5/5	N/N
4	41	2/2	1/1	N/A	N/A	N/Y
5	43	1/1	4/4	1/1	4-/4-	N/Y
6	41	1/1	4/4	4/4	5/5	N/N
7	34	0/0	0/0	4/4	5/5	N/N
8	51	0/0	1/1	3/3	3/3	Y/Y
9	48	N/A	N/A	N/A	N/A	N/A
10	53	1/1	4/4-	3/3	4/4	N/N
11	46	0/0	0/0	3/4	4/4	N/Y
12	44	4/4	4/4	5/5	5/5	N/N
13 (i)	48	5/5	5/5	5/5	5/5	N/N
13 (ii)	43	5/5	5/5	5/5	5/5	N/N
13 (iii)	62	3/3	3/3	N/A	N/A	N/Y
13 (iv)	48	0/0	0/0	4/4	4/4	N/Y
14 (i)	61	0/0	1/1	2/2	3/4	N/Y
14 (ii)	25	3/4	4/4	3/4	4/4	N/N
14 (iii)	27	1/1	4-/4-	4-/3	4/4	N/N

Roman numerals in parenthesis refer to family members. Numbers are Medical Research Council grades for power (right/left), NA = not available, ADF = ankle dorsiflexion, APF = ankle plantar flexion, FDIO = first dorsal interosseous, APB = abductor pollicis brevis, UL = upper limb, LL = lower limb, Y = yes, N = no.

**Table 3:** A summary of the natural history of patients with mutations in *HSPB1* as defined by the CMT examination score (CMTES), parenthesis = weighted CMTES score.

Patient	Year				
	0	1	2	3	4
1 (ii)	17(21)	12(17)	14(19)	17(23)	16(22)
8	9(13)	10(15)			
11	15(18)	7(9)	8(11)		
12	2(3)	4(7)	2(3)	3(5)	
13(i)	5(6)	9(13)	8(12)	9(13)	
13(ii)	0	0	0		
13(iv)	16(20)	14(17)	15(18)	15(19)	
14(ii)	9(13)	8(11)	9(13)		

## Appendix Supplementary material

Table S1 A summary of the nerve conduction parameters of patients with mutations in HSPB1 collected both retrospectively and prospectively over a 10 year period. Age = age at first examination, CMAP = compound muscle action potential, SAP = sensory action potential, F2 = 2nd finger, TA = tibialis anterior, EDB = extensor digitorum brevis, NR = not recordable. Table S1
