# Supplementary material for: Pilot phenotype and natural history study of hereditary neuropathies caused by mutations in the HSPB1 gene
Source: Neuromuscul Disord. 2017 Jan;27(1):50–6. doi: 10.1016/j.nmd.2016.10.001 (PMC5260843; doi:10.1016/j.nmd.2016.10.001)
Supplement: Table S1 — A summary of the nerve conduction parameters of patients with mutations in HSPB1 collected both retrospectively and prospectively over a 10 year period. Age = age at first examination, CMAP = compound muscle action potential, SAP = sensory action potential, F2 = 2nd finger, TA = tibialis anterior, EDB = extensor digitorum brevis, NR = not recordable. [file mmc1.docx]

| Patient | Age | Nerve | Year | | | | | | | | |
| --- | --- | --- | --- | --- | --- | --- | --- | --- | --- | --- | --- |
|  |  |  | 0 | 1 | 2 | 3 | 4 | 5 | 6 | 7 | 10 |
| 1 (i) | 79 | Median CMAP (wrist) | 4.8 |  |  |  |  |  |  |  |  |
|  |  | Ulnar CMAP (wrist) | 4.2 |  |  |  |  |  |  |  |  |
|  |  | Peroneal CMAP (TA) | 2.3 |  |  |  |  |  |  |  |  |
|  |  | Tibial CMAP | NR |  |  |  |  |  |  |  |  |
|  |  | Radial SAP | 1.9 |  |  |  |  |  |  |  |  |
|  |  | Median SAP | 2 |  |  |  |  |  |  |  |  |
|  |  | Sural SAP | 2 |  |  |  |  |  |  |  |  |
| 1 (ii) | 62 | Median CMAP (wrist) | 8.5 |  |  |  |  |  |  | 3.9 |  |
|  |  | Ulnar CMAP (wrist) | 7.2 |  |  |  |  |  |  | 5.4 |  |
|  |  | Peroneal CMAP (TA) | 3.8 |  |  |  |  |  |  |  |  |
|  |  | Tibial CMAP | NR |  |  |  |  |  |  |  |  |
|  |  | Radial SAP | 9.5 |  |  |  |  |  |  | 9 |  |
|  |  | Median SAP | 2.3 |  |  |  |  |  |  | 2 |  |
|  |  | Sural SAP | NR |  |  |  |  |  |  |  |  |
| 2 | 46 | Median CMAP (wrist) | 5.8 |  |  |  |  |  |  |  |  |
|  |  | Ulnar CMAP (wrist) | 9.2 |  |  |  |  |  |  |  |  |
|  |  | Peroneal CMAP (TA) | 4.2 |  |  |  |  |  |  |  |  |
|  |  | Tibial CMAP | 0.3 |  |  |  |  |  |  |  |  |
|  |  | Radial SAP | - |  |  |  |  |  |  |  |  |
|  |  | Median SAP | 12 |  |  |  |  |  |  |  |  |
|  |  | Sural SAP | 14 |  |  |  |  |  |  |  |  |
| 3 | 68 | Median CMAP (wrist) | 3.5 |  |  |  |  |  |  |  |  |
|  |  | Ulnar CMAP (wrist) | 4.5 |  |  |  |  |  |  |  |  |
|  |  | Peroneal CMAP (TA) | 1.3 |  |  |  |  |  |  |  |  |
|  |  | Tibial CMAP | NR |  |  |  |  |  |  |  |  |
|  |  | Radial SAP | 4 |  |  |  |  |  |  |  |  |
|  |  | Median SAP | 3 |  |  |  |  |  |  |  |  |
|  |  | Sural SAP | NR |  |  |  |  |  |  |  |  |
| 4 | 41 | Median CMAP (wrist) | 5.3 |  |  |  |  |  |  | 5.2 |  |
|  |  | Ulnar CMAP (wrist) | 6.4 |  |  |  |  |  |  | 4.3 |  |
|  |  | Peroneal CMAP (TA) | NR |  |  |  |  |  |  | 0.1 |  |
|  |  | Tibial CMAP | NR |  |  |  |  |  |  | NR |  |
|  |  | Radial SAP | 15 |  |  |  |  |  |  | 21 |  |
|  |  | Median SAP | 8 |  |  |  |  |  |  | 7 |  |
|  |  | Sural SAP | 3 |  |  |  |  |  |  | 5 |  |
| 5 | 43 | Median CMAP (wrist) | 5.8 |  |  |  |  |  | 0.3 |  |  |
|  |  | Ulnar CMAP (wrist) | 0.5 |  |  |  |  |  | 0.4 |  |  |
|  |  | Peroneal CMAP (TA) |  |  |  |  |  |  | 0.1 |  |  |
|  |  | Tibial CMAP |  |  |  |  |  |  | 0.1 |  |  |
|  |  | Radial SAP |  |  |  |  |  |  | - |  |  |
|  |  | Median SAP | 33 |  |  |  |  |  | 23 |  |  |
|  |  | Sural SAP | 18 |  |  |  |  |  | 8 |  |  |
| 6 | 41 | Median CMAP (wrist) | 9.7 |  |  |  |  |  |  |  |  |
|  |  | Ulnar CMAP (wrist) |  |  |  |  |  |  |  |  |  |
|  |  | Peroneal CMAP (EDB) | NR |  |  |  |  |  |  |  |  |
|  |  | Tibial CMAP | NR |  |  |  |  |  |  |  |  |
|  |  | Radial SAP |  |  |  |  |  |  |  |  |  |
|  |  | Median SAP | 13 |  |  |  |  |  |  |  |  |
|  |  | Sural SAP | 8 |  |  |  |  |  |  |  |  |
| 7 | 34 | Median CMAP (wrist) | 8.1 |  |  |  |  |  |  |  |  |
|  |  | Ulnar CMAP (wrist) | 0.9 |  |  |  |  |  |  |  |  |
|  |  | Peroneal CMAP (EDB) | 0.2 |  |  |  |  |  |  |  |  |
|  |  | Tibial CMAP | NR |  |  |  |  |  |  |  |  |
|  |  | Radial SAP | 40 |  |  |  |  |  |  |  |  |
|  |  | Median SAP | 7 |  |  |  |  |  |  |  |  |
|  |  | Sural SAP | 13 |  |  |  |  |  |  |  |  |
| 8 | 51 | Median CMAP (wrist) | 6.3 |  |  |  |  |  | 5.4 |  |  |
|  |  | Ulnar CMAP (wrist) | 5.0 |  |  |  |  |  | 2.2 |  |  |
|  |  | Peroneal CMAP (TA) | 0.2 |  |  |  |  |  | 0.0 |  |  |
|  |  | Tibial CMAP | NR |  |  |  |  |  |  |  |  |
|  |  | Radial SAP | 22 |  |  |  |  |  | 23 |  |  |
|  |  | Median SAP | 10 |  |  |  |  |  | 7 |  |  |
|  |  | Sural SAP | 8 |  |  |  |  |  | 6 |  |  |

| 9 | 48 | Median CMAP (wrist) | 7.6 |  |  |  |  |  |  |  |  |
| --- | --- | --- | --- | --- | --- | --- | --- | --- | --- | --- | --- |
|  |  | Ulnar CMAP (wrist) | 3.0 |  |  |  |  |  |  |  |  |
|  |  | Peroneal CMAP (EDB) | 1.0 |  |  |  |  |  |  |  |  |
|  |  | Tibial CMAP |  |  |  |  |  |  |  |  |  |
|  |  | Radial SAP |  |  |  |  |  |  |  |  |  |
|  |  | Median SA | 24 |  |  |  |  |  |  |  |  |
|  |  | Sural SAP | 13 |  |  |  |  |  |  |  |  |
| 10 | 53 | Median CMAP (wrist) | 3.3 | 3.6 |  |  |  |  |  |  |  |
|  |  | Ulnar CMAP (wrist) | 1.7 | 0.8 |  |  |  |  |  |  |  |
|  |  | Peroneal CMAP (TA) | 0.5 |  |  |  |  |  |  |  |  |
|  |  | Tibial CMAP | NR | NR |  |  |  |  |  |  |  |
|  |  | Radial SAP | 41 | 36 |  |  |  |  |  |  |  |
|  |  | Median SAP | 4 | 3 |  |  |  |  |  |  |  |
|  |  | Sural SAP | 12 | 1 |  |  |  |  |  |  |  |
| 11 | 46 | Median CMAP (wrist) | 10.9 |  |  | 7.8 |  |  |  |  | 7.0 |
|  |  | Ulnar CMAP (wrist) |  |  |  | 6.7 |  |  |  |  | 3.4 |
|  |  | Peroneal CMAP (TA) | 3.1 |  |  | 2.6 |  |  |  |  | 0.1 |
|  |  | Tibial CMAP | NR |  |  | NR |  |  |  |  | NR |
|  |  | Radial SAP |  |  |  |  |  |  |  |  | 34 |
|  |  | Median SAP | 27 |  |  | 25 |  |  |  |  | 13 |
|  |  | Sural SAP | 14.5 |  |  | 13 |  |  |  |  | 17 |
| 12 | 44 | Median CMAP (wrist) | 8.6 |  | 10.2 |  |  |  |  |  |  |
|  |  | Ulnar CMAP (wrist) | 7.9 |  |  |  |  |  |  |  |  |
|  |  | Peroneal CMAP (EDB) | 0.4 |  | 0.4 |  |  |  |  |  |  |
|  |  | Tibial CMAP |  |  |  |  |  |  |  |  |  |
|  |  | Radial SAP |  |  | 37 |  |  |  |  |  |  |
|  |  | Median SAP | 21 |  | 15 |  |  |  |  |  |  |
|  |  | Sural SAP | 13 |  | 9 |  |  |  |  |  |  |
| 13 (i) | 48 | Median CMAP (wrist) | 7.6 |  | 7.3 |  |  |  |  |  |  |
|  |  | Ulnar CMAP (wrist) | 10.0 |  | 9.8 |  |  |  |  |  |  |
|  |  | Peroneal CMAP (TA) | 3.4 |  | 5.0 |  |  |  |  |  |  |
|  |  | Tibial CMAP | NR |  | NR |  |  |  |  |  |  |
|  |  | Radial SAP | 26 |  | 21 |  |  |  |  |  |  |
|  |  | Median SAP | 6 |  | 4 |  |  |  |  |  |  |
|  |  | Sural SAP | 2 |  | 3 |  |  |  |  |  |  |
| 13 (ii) | 43 | Median CMAP (wrist) | 10.5 | 9.8 |  |  |  |  |  |  |  |
|  |  | Ulnar CMAP (wrist) | 9.9 | 10.8 |  |  |  |  |  |  |  |
|  |  | Peroneal CMAP (TA) |  |  |  |  |  |  |  |  |  |
|  |  | Tibial CMAP | 1.3 | 5.7 |  |  |  |  |  |  |  |
|  |  | Radial SAP | 31 | 46 |  |  |  |  |  |  |  |
|  |  | Median SAP | 18 | 18 |  |  |  |  |  |  |  |
|  |  | Sural SAP | 9 | 6 |  |  |  |  |  |  |  |
| 13 (iii) | 60 | Median CMAP (wrist) | 7.9 |  |  |  |  |  |  |  |  |
|  |  | Ulnar CMAP (wrist) |  |  |  |  |  |  |  |  |  |
|  |  | Peroneal CMAP (TA) |  |  |  |  |  |  |  |  |  |
|  |  | Tibial CMAP | NR |  |  |  |  |  |  |  |  |
|  |  | Radial SAP |  |  |  |  |  |  |  |  |  |
|  |  | Median SAP | 5 |  |  |  |  |  |  |  |  |
|  |  | Sural SAP | NR |  |  |  |  |  |  |  |  |
| 13 (iv) | 48 | Median CMAP (wrist) | 1.9 |  |  | 2.0 | 2.9 |  |  |  |  |
|  |  | Ulnar CMAP (wrist) | 3.3 |  |  | 4.4 | 4.7 |  |  |  |  |
|  |  | Peroneal CMAP (TA) | 0.1 |  |  |  |  |  |  |  |  |
|  |  | Tibial CMAP | NR |  |  |  |  |  |  |  |  |
|  |  | Radial SAP | 12 |  |  |  | 18 |  |  |  |  |
|  |  | Median SAP | 3 |  |  | 1 | 2 |  |  |  |  |
|  |  | Sural SAP | NR |  |  |  |  |  |  |  |  |
| 14 (i) | 61 | Median CMAP (wrist) | 4.0 |  |  |  |  |  |  |  |  |
|  |  | Ulnar CMAP (wrist) | 6.4 |  |  |  |  |  |  |  |  |
|  |  | Peroneal CMAP (TA) |  |  |  |  |  |  |  |  |  |
|  |  | Tibial CMAP | NR |  |  |  |  |  |  |  |  |
|  |  | Radial SAP | 16 |  |  |  |  |  |  |  |  |
|  |  | Median SAP | 8 |  |  |  |  |  |  |  |  |
|  |  | Sural SAP | 1 |  |  |  |  |  |  |  |  |

| 14 (ii) | 25 | Median CMAP (wrist) | 9.5 |  |  | 7.8 |  |  |  | 7.6 |  |
| --- | --- | --- | --- | --- | --- | --- | --- | --- | --- | --- | --- |
|  |  | Ulnar CMAP (wrist) | 7.8 |  |  | 7.8 |  |  |  | 7.8 |  |
|  |  | Peroneal CMAP (EDB) | 2.5 |  |  | 2.5 |  |  |  |  |  |
|  |  | Tibial CMAP | 4.7 |  |  |  |  |  |  |  |  |
|  |  | Radial SAP |  |  |  |  |  |  |  | 61 |  |
|  |  | Median SAP | 21 |  |  | 26 |  |  |  | 13 |  |
|  |  | Sural SAP | 20 |  |  | 15 |  |  |  | 8 |  |
| 14 (iii) | 27 | Median CMAP (wrist) | 7.3 |  |  |  |  | 2.9 |  |  |  |
|  |  | Ulnar CMAP (wrist) | 5.6 |  |  |  |  | 5.6 |  |  |  |
|  |  | Peroneal CMAP (EDB) | 1.8 |  |  |  |  | 1.6 |  |  |  |
|  |  | Tibial CMAP |  |  |  |  |  | 1.6 |  |  |  |
|  |  | Radial SAP | 58 |  |  |  |  | 50 |  |  |  |
|  |  | Median SAP | 31 |  |  |  |  | 24 |  |  |  |
|  |  | Sural SAP | 14 |  |  |  |  | 14 |  |  |  |

Supplementary Table 1. A summary of the nerve conduction parameters of patients with mutations in HSPB1 collected both retrospectively and prospectively over a 10 year period. Age = age at first examination, CMAP = compound muscle action potential, SAP = sensory action potential, F2 = 2nd finger, TA = tibialis anterior, EDB = extensor digitorum brevis, NR = not recordable.
